# Sarcopenia in patients with active ulcerative colitis: associations with serum metabolites and gut microbiota

**DOI:** 10.3389/fnut.2026.1942104

**Published:** 2026-09-03

**Authors:** Wei Wei, Pengguang Yan, Fang Wang, Qiong Wang, Chunwei Li, Xiaoyin Bai, Yu Zhang, Yuanyuan Bao, Jingnan Li, Kang Yu

**Affiliations:** 1 Department of Clinical Nutrition, Peking Union Medical College Hospital, Chinese Academy of Medical Sciences and Peking Union Medical College, Beijing, China; 2 Department of Gastroenterology, Peking Union Medical College Hospital, Chinese Academy of Medical Sciences and Peking Union Medical College, Beijing, China

**Keywords:** sarcopenia, ulcerative colitis, gut microbiota, metabolites, systemic inflammation

## Abstract

**Background:**

Sarcopenia has garnered increasing attention in ulcerative colitis (UC) owing to its association with adverse clinical outcomes; however, its pathogenesis in the context of UC remains insufficiently characterized.

**Methods:**

Hospitalized patients aged 18–70 years with active UC were consecutively enrolled at the Department of Gastroenterology, Peking Union Medical College Hospital, and age-matched healthy controls (HCs) were recruited. Body composition was assessed by bioelectrical impedance analysis (BIA), and muscle strength was evaluated by handgrip strength. Sarcopenia was diagnosed in accordance with the Asian Working Group for Sarcopenia (AWGS) 2025 consensus. Serum high-sensitivity C-reactive protein (hsCRP) of patients was recorded at admission. Intestinal barrier function was evaluated by serum diamine oxidase (DAO). Serum metabolites were profiled by ultra-performance liquid chromatography–tandem mass spectrometry (UPLC-MS/MS). Gut microbiota composition and functional pathways were analyzed using metagenomic sequencing.

**Results:**

Fifty-seven patients with active UC and 35 HCs were enrolled. The prevalence of sarcopenia, myopenia, and low muscle strength in the UC cohort was 40.4, 50.9, and 64.9%, respectively. Serum hsCRP was significantly higher in UC patients with sarcopenia (*p* = 0.011), whereas serum DAO showed no significant difference between UC patients with and without sarcopenia. Primary bile acids (BAs), conjugated BAs, and conjugated primary BAs were significantly increased, while hippuric acid, valerylcarnitine, and 2-methylbutyrylcarnitine were significantly decreased, in sarcopenic relative to non-sarcopenic UC patients (all *p* < 0.05). However, the association between serum hippuric acid and sarcopenia was not significant after adjusting for disease activity and hsCRP. At the genus level, *Bacteroides* and *Streptococcus* were the most prominently decreased and increased taxa in sarcopenic UC patients, respectively. Metagenomic functional analysis revealed that the relative abundance of the protein digestion and absorption pathway was significantly lower in sarcopenic UC patients (*p* = 0.030).

**Conclusion:**

UC-related sarcopenia exhibits notable associations with systemic inflammation, gut dysbiosis, and perturbations in circulating metabolites, including BAs and acylcarnitines.

## 1 Introduction

Ulcerative colitis (UC), a major subtype of inflammatory bowel disease (IBD), is a chronic, relapsing–remitting inflammatory disorder characterized by continuous and diffuse mucosal inflammation affecting the rectum and colon. Over the past three decades, the burden of IBD in China has risen substantially and is projected to increase further by 2030 (1). The peak age of UC onset in the Chinese population falls between 50 and 59 years (2). Despite rapid advances in therapies for UC, mainly in biologics and small molecules, remission rates do not surpass 20–30% in induction clinical trials and 30–60% in a real-life setting, leading to the requirement of surgery for medically refractory disease (3). The etiology of IBD involves complex interactions among genetic susceptibility, environmental triggers, immune dysregulation, and gut dysbiosis (4, 5), culminating in chronic intestinal inflammation, epithelial barrier disruption, and malabsorption (6). A network of pro-inflammatory cytokines produced by diverse inflammatory cells contributes to the pathology of UC, such as tumor necrosis factor-α, interleukin (IL)-6, IL-1β, IL-12/23, and IL-18 (7).

Currently, the diagnosis of sarcopenia in UC relies primarily on criteria established by the European Working Group on Sarcopenia in Older People (EWGSOP) or the Asian Working Group for Sarcopenia (AWGS), due to the lack of specific criteria for disease-related sarcopenia (8). According to a 2023 systematic review, the prevalence of sarcopenia and myopenia in UC patients was estimated at 14 and 43%, respectively (9). Previous research has demonstrated a stepwise increase in sarcopenia with worsening disease severity in UC patients (10). Accumulating evidence supports that sarcopenia may increase the risk of overall treatment failure and the need for surgical intervention in UC patients (11). Furthermore, sarcopenia has been associated with an elevated risk of postoperative complications in patients with acute severe UC (12).

The term “gut–muscle axis” was first proposed by Ticinesi et al. in 2017 (13) and has since emerged as a novel framework for understanding the complex bidirectional communication between the gastrointestinal system and skeletal muscle. A recent bidirectional Mendelian randomization study demonstrated that the gut microbiome mediates the effect of IBD—principally Crohn’s disease—on sarcopenia-related traits, including reduced handgrip strength and appendicular lean mass (14). Moreover, integrative bioinformatics analysis by Deng et al. identified shared immune–metabolic transcriptomic features and potential diagnostic biomarkers between UC and sarcopenia, highlighting fatty acid metabolism-related hub genes PHYH and HSD17B3 (15). In older adults affected by physical frailty and sarcopenia, the gut microbiota community characterized by increased *Oscillospira* and *Ruminococcus* as well as decreased Barnesiellaceae and Christensenellaceae has been reported (16). Furthermore, a randomized clinical trial showed that probiotic supplementation (*Lactobacillus paracasei* strains) improved amino acid absorption from plant protein, increasing the blood levels of total essential amino acids and total branched-chain amino acids (BCAAs) (17). With respect to metabolites, altered short-chain fatty acid (SCFA) metabolism has been observed in elderly Chinese women with sarcopenia, evidenced by decreased serum propionic acid and isovaleric acid, and increased serum caproic acid (18). However, in contrast with the research in the field of age-related sarcopenia, data on the microbial signatures and metabolic pathways linking UC and sarcopenia remain limited, and previous studies on UC and sarcopenia have predominantly focused on the relationship between sarcopenia and clinical outcomes rather than on the pathogenesis of sarcopenia in UC.

Therefore, the present study aimed to explore the prevalence of sarcopenia in a cohort of hospitalized patients with active UC using the updated cut-off values from the AWGS 2025 consensus, and to investigate the associations between intestinal barrier function, systemic inflammation, circulating metabolites, gut microbiota composition, and sarcopenia in this population.

## 2 Materials and methods

### 2.1 Study participants

Hospitalized patients aged 18 to 70 years with active UC at the Department of Gastroenterology, Peking Union Medical College Hospital (PUMCH), were consecutively enrolled from November 2022 to June 2023. All patients were diagnosed with UC according to the European Crohn’s and Colitis Organisation (ECCO) consensus guidelines (19), based on clinical manifestations, endoscopic findings, and histopathologic characteristics, with infectious colitis and other non-infectious colitis excluded. Patients were excluded if they (1) exhibited laboratory evidence of impaired hepatic or renal function; (2) had comorbidities involving other major organs (e.g., cardiovascular, pulmonary, hepatic, or renal) or endocrine, hematological, or autoimmune diseases other than UC; (3) had neuromuscular or orthopedic conditions; (4) had a history of malignancy or cancer-related comorbidity; or (5) were pregnant or lactating.

Age-matched healthy controls (HCs) were recruited from May to June 2023. Inclusion criteria were as follows: (1) age ≥ 18 and ≤ 70 years; (2) no history of major medical conditions, including hepatic, gastrointestinal, cardiac, respiratory, renal, endocrine, hematological, autoimmune, neuromuscular, or orthopedic diseases; (3) laboratory tests within the preceding year showing no abnormalities in complete blood count, hepatic or renal function, lipid profile, or fasting glucose; and (4) no use of microbiota-modulating agents (e.g., probiotics, prebiotics), antibiotics, antidepressants, or nonsteroidal anti-inflammatory drugs (NSAIDs) within the preceding 2 weeks, nor glucocorticoids, immunosuppressants, lipid-lowering agents, or hypoglycemic agents within the preceding 3 months. Subjects with low muscle mass or low muscle strength based on the criteria in AWGS 2025 were excluded (20), and pregnant or lactating women were excluded.

Informed consent was obtained from each participant prior to study entry. The study was approved by the Ethics Committee of PUMCH (No. I-22PJ700) and was conducted in compliance with the Declaration of Helsinki.

### 2.2 Collection of clinical data

Clinical data of UC patients, including disease duration, disease activity, and values of hemoglobin (HGB), serum albumin (ALB), and hsCRP at admission, were extracted from medical records. Disease activity was assessed according to the modified Truelove and Witts’ criteria.

### 2.3 Assessment of body composition and diagnosis of sarcopenia

All participants underwent body composition measurement using the multi-frequency bioimpedance analyzer InBody S10 (Biospace Co., Ltd., Korea). Measurements were performed at the bedside upon admission for patients and at the Department of Clinical Nutrition for HCs. Bioelectrical impedance analysis (BIA) parameters included fat-free mass (FFM), fat mass (FM), FM percentage (FM%), regional skeletal muscle mass (trunk and limbs), visceral fat area (VFA), extracellular water-to-total body water ratio (ECW/TBW), and bone mineral content (BMC). The appendicular skeletal muscle index (ASMI) and trunk skeletal muscle index (TSMI) were derived by normalizing appendicular and trunk muscle mass to height squared (m^2^), respectively. The BIA-derived phase angle (PhA) was measured at 50 kHz and expressed in degrees. Additionally, calf circumference (CC) was measured at the maximal calf girth using a non-elastic tape (0.1 cm precision).

In parallel, for patients who underwent abdominal computed tomography (CT) for disease evaluation within one week before or after admission, skeletal muscle area (SMA), VFA, and subcutaneous fat area (SFA) were quantified at the third lumbar vertebra (L3) cross-section, as previously described (21). The skeletal muscle index (SMI) was derived by normalizing SMA to height squared (m^2^).

Muscle strength was assessed by handgrip strength of the dominant hand using a calibrated electronic dynamometer, with a uniform handle setting applied to all participants. Three maximal voluntary contractions were performed, and the maximum value (in kilograms) was recorded.

Sarcopenia was diagnosed in accordance with the AWGS 2025 consensus (20). For patients aged 65–70 years, low muscle strength was defined as handgrip strength < 28 kg in males and < 18 kg in females, and myopenia was defined as ASMI < 7.0 kg/m^2^ in males and < 5.7 kg/m^2^ in females, consistent with the AWGS 2019 consensus values (22). For patients aged 18–64 years, the cut-off values were derived from those established for the population aged 50–64 years in AWGS 2025: low muscle strength was defined as handgrip strength < 34 kg in males and < 20 kg in females, and myopenia was defined as ASMI < 7.6 kg/m^2^ in males and < 5.7 kg/m^2^ in females. Sarcopenia was diagnosed based on the coexistence of low muscle strength and myopenia.

### 2.4 Assessment of intestinal barrier function

Fasting blood samples were collected from each patient; serum aliquots were prepared within 1 h and stored at −80 °C until analysis. Serum DAO concentration was determined using an ELISA kit (Cloud-Clone Corp., China) according to the manufacturer’s instructions.

### 2.5 Targeted serum metabolomic analysis

Serum metabolites were measured for each participant, including amino acids, bile acids (BAs), carnitines, SCFAs, benzoic acids, and indoles. The specific metabolites analyzed are listed in Supplementary Table 1. All standards of the targeted metabolites were obtained from Sigma-Aldrich (USA), Steraloids Inc. (USA), and TRC Chemicals (Canada). An ultra-performance liquid chromatography coupled to tandem mass spectrometry (UPLC-MS/MS) system (ACQUITY UPLC-Xevo TQ-S, Waters Corp., USA) was employed to quantify all targeted metabolites. To minimize sample degradation, serum samples were initially thawed on an ice bath and subsequently extracted using methanol. Then, samples were injected into an ACQUITY UPLC BEH C18 1.7 μm VanGuard pre-column (2.1 × 5 mm) and an ACQUITY UPLC BEH C18 1.7 μm analytical column (2.1 × 100 mm). Details of parameter settings are shown in Supplementary Table 2. Raw data files acquired by UPLC-MS/MS were processed using MassLynx software (v4.1, USA) for peak integration, calibration, and quantitation.

### 2.6 Metagenomic sequencing and microbial community function annotation

Fresh stool samples were collected in sterile plastic tubes from all participants, immediately transferred to the laboratory, and stored at −80 °C until analysis. DNA extraction was performed using the QIAamp Fast DNA Stool Mini Kit (Germany) according to the manufacturer’s instructions. DNA libraries with approximately 500 bp insert sizes were constructed following the Illumina TruSeq DNA Sample Prep v2 Guide (Illumina, Inc., USA). Quality control of all libraries was performed using the Agilent 2,100 Bioanalyzer (Agilent Technologies, UK). Metagenomic sequencing was carried out on the HiSeq X-Ten platform (Illumina, Inc., USA) with 150-bp paired-end read length.

Illumina raw reads were filtered by removing adaptor-contaminated reads, reads containing more than three ambiguous N bases, reads with low-quality (Q < 20) bases, and reads with fewer than 60% high-quality bases (Phred score ≥ 20). Clean reads were aligned to bacterial genomes from the NCBI GenBank using SOAPaligner (version 2.21); reads mapping to the host genome were removed, and taxonomic relative abundance profiles were generated at various taxonomic levels. Alpha-diversity indices, including Shannon and Simpson indices, were analyzed to characterize microbial diversity and evenness. Comparisons of microbial community structure were conducted using Principal Coordinate Analysis (PCoA), Analysis of Similarities (ANOSIM), and Multi-Response Permutation Procedure (MRPP). Linear discriminant analysis (LDA) Effect Size (LEfSe) was employed to identify species with significant abundance differences between groups, applying cut-off values of |log10 LDA score| > 2.0 and *p* < 0.05.

Preprocessed reads were assembled using SOAPdenovo (version 1.05). Open reading frames (ORFs) were predicted using MetaGeneMark. A non-redundant gene catalog was constructed by pairwise comparison of predicted ORFs (gene length > 100 bp) using CD-HIT (version 4.5.7). Reads were subsequently mapped to the representative genes at a 95% identity threshold using SOAPaligner (version 2.21), and gene abundance in each sample was quantified. Using BLAST (version 2.2.28+), non-redundant genes were annotated against the Kyoto Encyclopedia of Genes and Genomes (KEGG) database. The relative abundance of each KEGG Orthology (KO) group was calculated, and KEGG enrichment analysis was subsequently performed to compare the levels of biologically relevant metabolic pathways between groups.

### 2.7 Statistical analysis

Demographic and clinical parameters are presented as mean ± standard deviation (SD) for normally distributed variables or median (Q1, Q3) for non-normally distributed variables. Between-group comparisons were performed using the independent-samples t-test or the nonparametric Mann–Whitney U-test for continuous variables, and the chi-squared test for categorical variables. A two-sided *p* < 0.05 was considered statistically significant. Correlations between body composition parameters measured by BIA and CT were evaluated using Pearson correlation coefficients for normally distributed pairs and Spearman rank-order correlation coefficients otherwise. Correlations between the relative abundance of gut microbial metabolic pathways and serum metabolite concentrations were evaluated using Spearman correlation. Statistical analyses above were performed using SPSS (version 23.0) and GraphPad Prism (version 8.0).

Multivariate logistic regression analysis was used to explore the associations between sarcopenia and gut microbiota or serum metabolites. Because of the limited sample size and quasi-complete separation for several variables, which yield extreme and unstable maximum-likelihood estimates, we used Bayesian logistic regression (23, 24) with each exposure (gut microbial taxon or serum metabolite) modeled separately, and all models were adjusted for hsCRP and disease activity. Weakly informative priors were adopted: independent Normal (0, 2.5) priors on the regression coefficients (log-OR scale) and a Normal (0, 5) prior on the intercept. The odds ratio (OR) represents the change in the odds of sarcopenia per 1-SD increase in the exposure. Models were fitted in R (version 4.6.1) using the rstanarm package (version 2.32.2). Four chains of 4,000 iterations (with the first 2000 discarded as warm-up) were run for each model, and convergence was assessed using the R-hat statistic (all R-hat < 1.01). Results were reported as the posterior median odds ratio with 95% credible intervals (CrI) (2.5th–97.5th posterior percentiles); two-sided Bayesian *p*-values were computed as 2 × min {*p* (OR < 1), *p* (OR > 1)}.

## 3 Results

### 3.1 Clinical characteristics and body composition assessments

A total of 57 patients with active UC (32 males, 25 females) and 35 HCs (18 males, 17 females) were enrolled. Among UC patients, 2 (3.5%, both female) had mild disease activity, 22 (38.6%, 13 males and 9 females) had moderate disease activity, and 33 (57.9%, 19 males and 14 females) had severe disease activity, with no significant sex difference in disease activity distribution (*p* = 0.399). Additionally, no significant differences were observed between male and female patients in age (45.8 ± 13.2 *vs*. 40.6 ± 11.5 years, *p* = 0.125) or disease duration [24 (9, 114) months *vs*. 48 (24, 126) months, *p* = 0.227]. Serum hsCRP at admission was comparable between male and female patients [14.13 (4.36, 28.34) mg/L in males *vs*. 16.30 (2.10, 35.02) mg/L in females, *p* = 0.923], indicating a similar inflammatory burden.

Demographic data and body composition parameters of UC patients and HCs are presented in Table 1. Compared with HCs, both male and female UC patients exhibited significantly lower body mass index (BMI) (*p* = 0.006 in males; *p* = 0.007 in females), FFM (*p* = 0.008 in males; *p* = 0.001 in females), FM (*p* = 0.021 in males; *p* = 0.037 in females), TSMI (*p* = 0.001 in males; *p* = 0.002 in females), CC (both *p* < 0.001 in males and females), PhA (both *p* < 0.001 in males and females), and handgrip strength (both *p* < 0.001 in males and females), as well as significantly higher ECW/TBW (*p* = 0.001 in males; *p* < 0.001 in females). ASMI was significantly lower in female patients relative to HCs (*p* < 0.001), whereas the difference between male patients and HCs did not reach statistical significance. FM% did not differ significantly between patients and HCs in either sex. Male patients showed a trend toward lower VFA compared with HCs (*p* = 0.064), while no significant difference was observed between female patients and HCs.

**Table 1:** Comparisons of body composition and muscle strength between ulcerative colitis patients and controls.

Variables	Males			Females		
	UC patients (*N* = 32)	Controls (*N* = 18)	*p* value	UC patients (*N* = 25)	Controls (*N* = 17)	*p* value
Age, years	45.8 ± 13.3	40.1 ± 9.8	0.115	40.5 ± 11.3	38.7 ± 11.3	0.612
BMI (kg/m^2^)	21.7 ± 3.9	24.3 ± 2.5	0.006	19.4 ± 2.8	21.7 ± 2.0	0.007
FFM (kg)	51.7 ± 7.5	57.2 ± 5.1	0.008	37.2 ± 3.5	41.1 ± 3.6	0.001
ASMI (kg/m^2^)	7.86 ± 1.20	8.14 ± 0.58	0.285	5.67 ± 0.57	6.42 ± 0.44	< 0.001
TSMI (kg/m^2^)	7.31 ± 1.11	8.19 ± 0.56	0.001	5.98 ± 1.00	6.75 ± 0.49	0.002
CC (cm)	34.7 ± 4.0	38.9 ± 2.3	< 0.001	32.1 ± 3.2	36.1 ± 1.6	< 0.001
FM (kg)	11.4 (8.0, 14.8)	16.8 (11.5, 23.0)	0.021	12.6 (9.5, 17.6)	17.2 (14.5, 19.7)	0.037
FM%	18.0 (13.7, 22.4)	22.7 (15.3, 29.8)	0.110	27.4 (20.2, 31.9)	29.4 (26.1, 31.6)	0.223
VFA (cm^2^)	43.4 (32.1, 65.8)	65.4 (40.0, 89.6)	0.064	59.8 (46.8, 85.3)	70.9 (58.2, 85.6)	0.481
BMC (kg)	2.86 ± 0.49	3.26 ± 0.37	0.004	2.31 ± 0.23	2.43 ± 0.25	0.123
ECW/TBW	0.387 ± 0.016	0.376 ± 0.007	0.001	0.393 ± 0.012	0.383 ± 0.004	< 0.001
PhA(°)	5.9 ± 1.9	7.5 ± 1.0	< 0.001	4.7 ± 1.3	6.8 ± 1.9	< 0.001
Handgrip Strength (kg)	32.0 ± 10.5	43.6 ± 7.3	< 0.001	18.7 ± 5.8	27.1 ± 3.4	< 0.001

The data are presented as mean ± SD or median (Q1, Q3). BMI, body mass index; FFM, fat-free mass; ASMI, appendicular skeletal muscle index; TSMI, trunk skeletal muscle index; CC, calf circumference; FM, fat mass; FM%, FM percentage; VFA, visceral fat area; BMC, bone mineral content; ECW/TBW, extracellular water-to-total body water ratio; PhA, phase angle.

### 3.2 Prevalence of sarcopenia in this UC cohort

Based on the AWGS 2025 criteria, 37 patients (64.9%) had low muscle strength [21 (65.6%) in males and 16 (64.0%) in females], and 29 patients (50.9%) had myopenia [14 (43.8%) in males and 15 (60.0%) in females]. In total, 23 patients (40.4%) met the criteria for sarcopenia [12 (37.5%) in males and 11 (44.0%) in females]. By comparison, when diagnosed according to the AWGS 2019 consensus (16), only 10 patients (17.5%; 1 male and 9 females) met the sarcopenia criteria, and the numbers of patients with low muscle strength or myopenia were both 20 (35.1%).

The comparison of muscle conditions between younger and older UC patients is presented in Table 2. The prevalence of sarcopenia and myopenia did not differ significantly between patients aged 18–49 years and those aged 50–70 years under either the AWGS 2025 or AWGS 2019 criteria. The prevalence of low muscle strength was significantly lower in younger patients than older patients under the AWGS 2019 criteria (25.6% *vs.* 64.3%, *p* = 0.021), whereas no significant difference was observed under the AWGS 2025 criteria. Furthermore, within younger patients, the prevalence of sarcopenia (44.2% *vs.* 18.6%, *p* = 0.011) and low muscle strength (65.1% *vs.* 25.6%, *p* < 0.001) was significantly higher when the AWGS 2025 criteria were applied.

**Table 2:** Comparison of muscle conditions in UC patients from different age groups.

Muscle conditions	UC patients aged 18–49 years (*N* = 43)	UC patients aged 50–70 years (*N* = 14)	*p* value
Sarcopenia			
AWGS 2025 criteria	19 (44.2%)	4 (28.6%)	0.301
AWGS 2019 criteria	8 (18.6%)	2 (14.3%)	1.000
Myopenia			
AWGS 2025 criteria	23 (53.5%)	6 (42.9%)	0.490
AWGS 2019 criteria	16 (37.2%)	4 (28.6%)	0.790
Low muscle strength			
AWGS 2025 criteria	28 (65.1%)	9 (64.3%)	1.000
AWGS 2019 criteria	11 (25.6%)	9 (64.3%)	0.021

The data are presented as *n* (%). AWGS, Asian Working Group for Sarcopenia.

**Figure 1:**
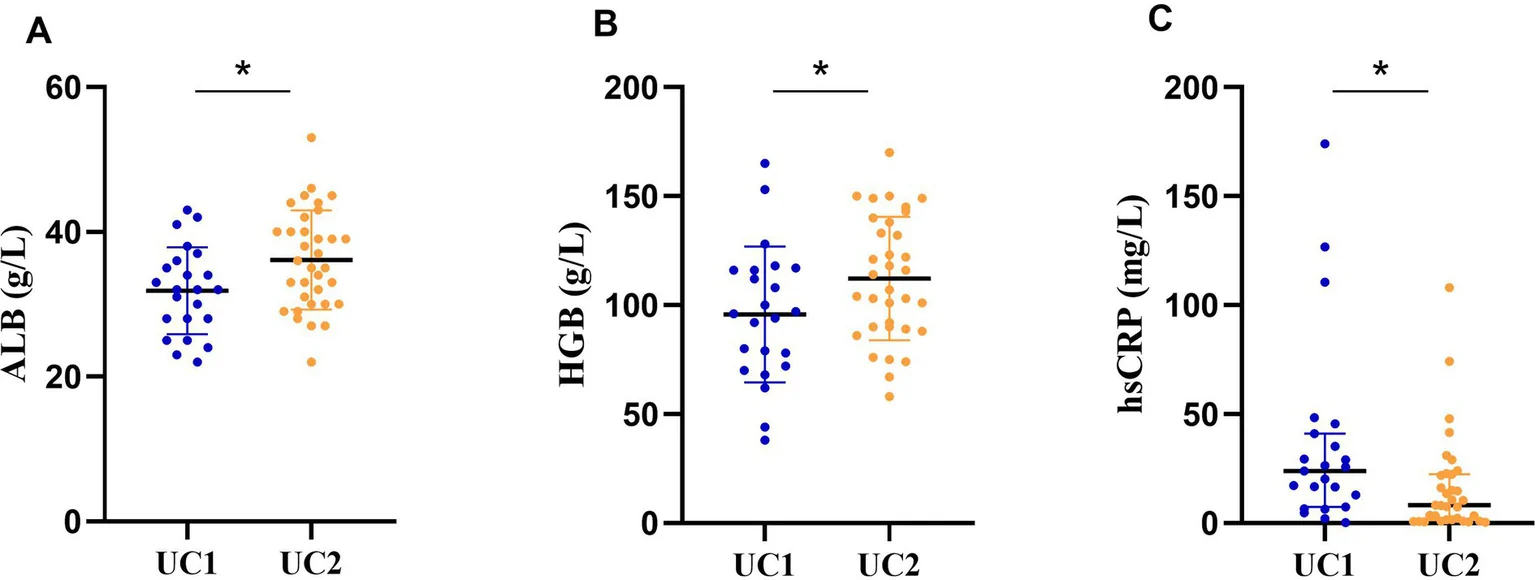
The comparison of laboratory indices between UC patients with and without sarcopenia. Horizontal lines in **(A,B)** indicate the mean and SD; horizontal lines in **(C)** indicate the median and interquartile range. HGB, hemoglobin; ALB, albumin; hsCRP, high-sensitivity C-reactive protein. UC1, UC patients with sarcopenia; UC2, UC patients without sarcopenia. *p < 0.05.

Demographic, clinical, and body composition data of sarcopenic and non-sarcopenic patients are presented in Table 3. No significant differences were observed in age, disease duration, fat indices, or PhA between the two groups. BMC was significantly lower in the sarcopenia group (*p* = 0.001). The proportion of patients with severe disease activity exhibited a higher trend in the sarcopenia group compared with the non-sarcopenia group (69.6% *vs.* 50.0%), but this difference was not statistically significant. The sarcopenia group had significantly higher hsCRP [23.90 (7.40, 41.03) mg/L *vs.* 8.18 (1.37, 22.48) mg/L, *p* = 0.011], and significantly lower HGB (95.8 ± 31.1 g/L *vs.* 112.3 ± 28.3 g/L, *p* = 0.043) and ALB (31.9 ± 6.0 g/L *vs.* 36.1 ± 6.8 g/L, *p* = 0.019) (Figures 1A–C).

**Table 3:** The demographics, clinical characteristics, and body composition of ulcerative colitis patients with and without sarcopenia.

Variables	Sarcopenia (*N* = 23)	Non-sarcopenia (*N* = 34)	*p* value
Age, years	43.7 ± 10.8	43.4 ± 13.9	0.938
Female (%)	11 (47.8%)	14 (41.2%)	0.620
BMI (kg/m^2^)	19.4 ± 2.8	21.6 ± 3.9	0.020
Disease duration, months	48 (10, 144)	36 (12, 90)	0.509
Patients with severe disease activity (%)	16 (69.6%)	17 (50.0%)	0.142
FFM (kg)	41.0 ± 7.2	48.3 ± 9.7	0.003
ASMI (kg/m^2^)	6.13 ± 0.94	7.42 ± 1.53	< 0.001
TSMI (kg/m^2^)	6.42 ± 1.21	6.93 ± 1.25	0.132
CC (cm)	31.8 ± 3.5	34.7 ± 3.6	0.004
FM (kg)	12.0 (8.1, 14.8)	11.7 (8.6, 16.9)	0.531
FM%	22.1 (15.6, 28.0)	20.8 (13.9, 23.0)	0.591
VFA (cm^2^)	53.1 (41.1, 73.6)	53.3 (32.2, 75.1)	0.795
BMC (kg)	2.40 ± 0.30	2.77 ± 0.53	0.001
ECW/TBW	0.393 ± 0.015	0.387 ± 0.015	0.169
PhA(°)	5.2 ± 1.9	5.4 ± 1.6	0.708
Handgrip Strength (kg)	21.1 ± 8.2	29.7 ± 11.3	0.003

The data are presented as mean ± SD, *n* (%), or median (Q1, Q3). BMI, body mass index; FFM, fat-free mass; ASMI, appendicular skeletal muscle index; TSMI, trunk skeletal muscle index; SMI, skeletal muscle index; CC, calf circumference; FM, fat mass; FM%, FM percentage; VFA, visceral fat area; BMC, bone mineral content; ECW/TBW, extracellular water-to-total body water ratio; PhA, phase angle.

### 3.3 Correlation analysis between body composition parameters measured by BIA and CT

**Figure 2:**
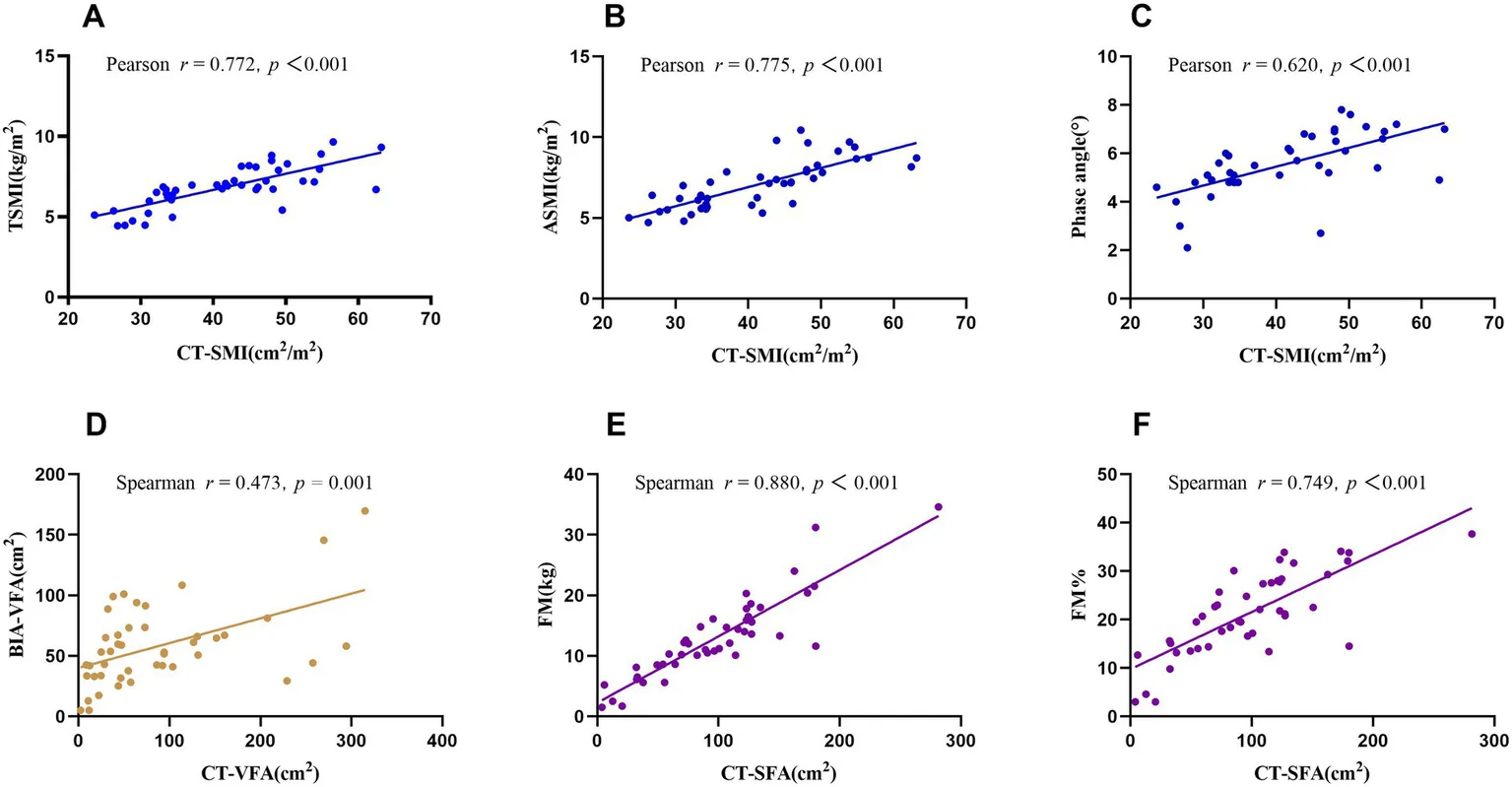
Correlations between body composition parameters measured by bioelectrical impedance analysis (BIA) and computed tomography (CT). Significant positive correlations were observed between CT-derived SMI and BIA-measured TSMI, ASMI, and phase angle **(A–C)**; CT-derived VFA and BIA-measured VFA also exhibited a significant positive correlation **(D)**, and CT-derived SFA showed significant positive correlations with BIA-measured FM and FM% **(E,F)**. SMI, skeletal muscle index; TSMI, trunk skeletal muscle index; ASMI, appendicular skeletal muscle index; VFA, visceral fat area; SFA, subcutaneous fat area; FM, fat mass; FM%, FM percentage.

Among the 57 enrolled patients, 44 underwent abdominal CT within one week before or after BIA measurement. Significant positive correlations were observed between CT-derived SMI and BIA-measured TSMI (*r* = 0.772, *p* < 0.001), ASMI (*r* = 0.775, *p* < 0.001), and PhA (*r* = 0.620, *p* < 0.001) (Figures 2A–C). Moreover, VFA values obtained by the two modalities exhibited a significant positive correlation (*r* = 0.473, *p* = 0.001) (Figure 2D), and CT-quantified SFA showed significant positive correlations with BIA-measured FM (*r* = 0.880, *p* < 0.001) and FM% (*r* = 0.749, *p* < 0.001) (Figures 2E,F).

### 3.4 Comparison of intestinal barrier function in UC patients with different muscle conditions

No significant difference in serum DAO was observed between sarcopenic and non-sarcopenic UC patients. However, among patients with myopenia, serum DAO tended to be higher than in those with normal muscle mass [0.814 (0.757, 0.877) ng/ml vs. 0.765 (0.739, 0.816) ng/ml, *p* = 0.057]. Similarly, patients with low muscle strength exhibited a trend toward elevated serum DAO relative to those with normal muscle strength [0.800 (0.756, 0.871) ng/ml vs. 0.751 (0.735, 0.803) ng/ml, *p* = 0.061].

### 3.5 Altered profile of serum metabolites in UC patients with sarcopenia

First, levels of serum metabolites in UC patients with and without sarcopenia were compared to identify potential distinguishing metabolites of UC-related sarcopenia. Regarding BAs, serum glycochenodeoxycholic acid-3-sulfate (GCDCA-3S; *p* < 0.001), glycocholic acid (GCA; *p* = 0.020), and taurocholic acid (TCA; *p* = 0.037) were significantly elevated in the sarcopenia group, while GCDCA showed an increasing trend (*p* = 0.074). Primary BAs (the total amount of cholic acid, chenodeoxycholic acid, and their glycine or taurine conjugated BAs; *p* = 0.029), conjugated BAs (the total amount of glycine or taurine conjugated BAs; *p* = 0.008), and conjugated primary BAs (*p* = 0.006) were all significantly increased in UC patients with sarcopenia, whereas secondary BAs did not differ significantly between groups. In contrast, hippuric acid—a benzoic acid derivative—was significantly decreased in the sarcopenia group (*p* = 0.002). Among carnitines, serum valerylcarnitine (*p* = 0.009) and 2-methylbutyrylcarnitine (*p* = 0.035) were also significantly decreased in the sarcopenia group, and isovalerylcarnitine exhibited a decreasing trend (*p* = 0.090). No significant differences were observed in serum levels of amino acids, SCFAs, or indoles between sarcopenic and non-sarcopenic UC patients.

**Figure 3:**
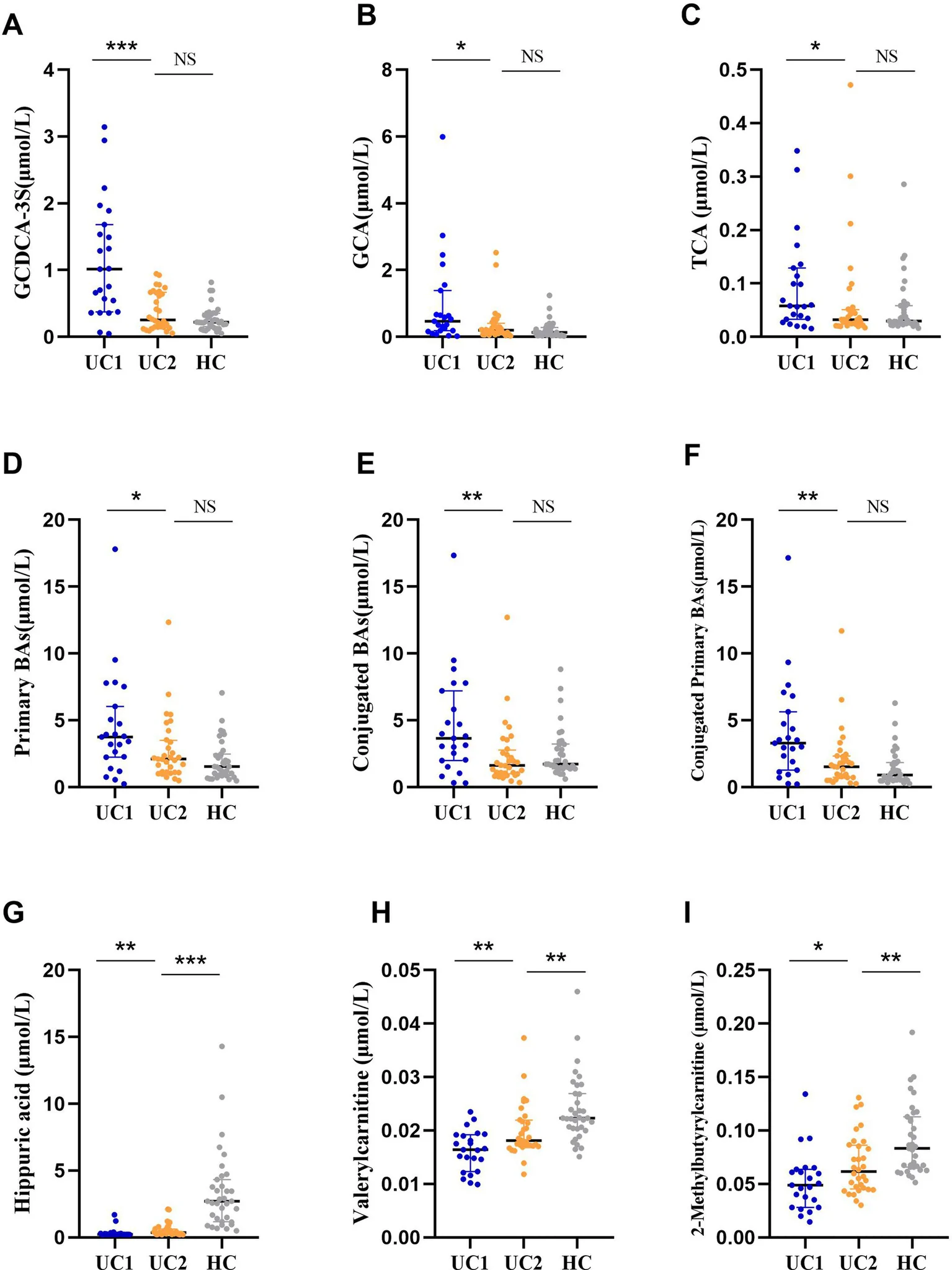
The comparisons of serum metabolites among sarcopenic UC patients, non-sarcopenic UC patients, and HCs. The serum concentrations of GCDCA-3S, GCA, TCA, primary BAs, conjugated BAs, and conjugated primary BAs were significantly higher in sarcopenic UC patients than those in non-sarcopenic UC patients **(A-F)**. Meanwhile, the serum concentrations of hippuric acid, valerylcarnitine, and 2-methylbutyrylcarnitine showed a stepwise increase among sarcopenic UC patients, non-sarcopenic UC patients, and HCs **(G-I)**. Horizontal lines indicate the median and interquartile range. UC, Ulcerative colitis; GCDCA-3S, Glycochenodeoxycholic acid-3-sulfate; GCA, Glycocholic acid; TCA, Taurocholic acid. UC1, UC patients with sarcopenia; UC2, UC patients without sarcopenia; HC, Healthy controls. *p < 0.05; **p < 0.01; ***p < 0.001; NS, Not significant.

Then, the levels of serum metabolites mentioned above were compared between HCs and UC patients without sarcopenia. Hippuric acid (*p* < 0.001), valerylcarnitine (*p* = 0.002), and 2-methylbutyrylcarnitine (*p* = 0.002) were all significantly lower in non-sarcopenic UC patients compared with HCs. However, among BAs, none of GCDCA-3S, GCA, TCA, primary BAs, conjugated BAs, and conjugated primary BAs showed significant differences between the two groups. The comparisons of these metabolites among sarcopenic UC patients, non-sarcopenic UC patients, and HCs are shown in Figure 3.

### 3.6 Altered gut microbiota of UC patients with sarcopenia

**Figure 4:**
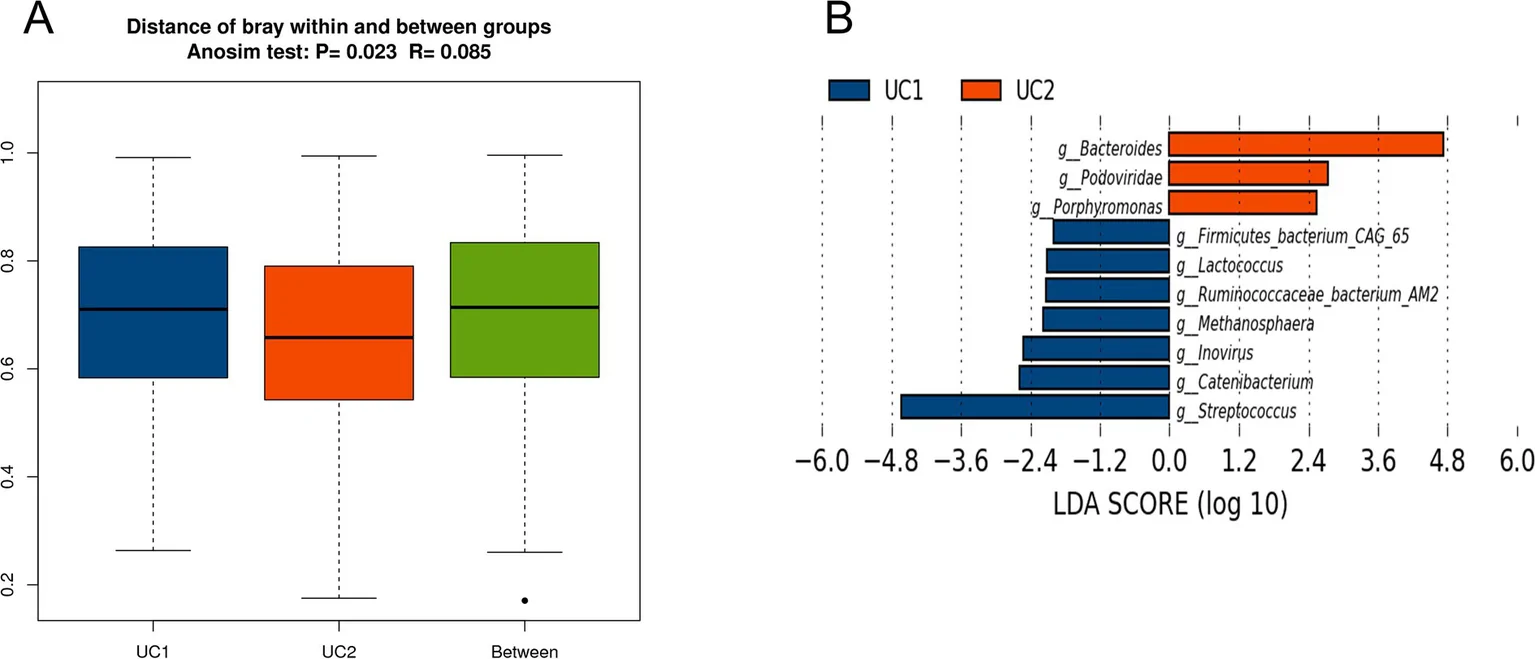
Altered gut microbiota composition in sarcopenic UC patients. **(A)** The result of Analysis of Similarities (ANOSIM). **(B)** The distinguishing taxa at genus level between UC patients with sarcopenia and without sarcopenia. UC1, UC patients with sarcopenia; UC2, UC patients without sarcopenia.

Alpha-diversity of the gut microbiota did not differ significantly between UC patients with and without sarcopenia. Although PCoA did not reach statistical significance, both ANOSIM (R = 0.085, *p* = 0.023; Figure 4A) and MRPP (A = 0.012, *p* = 0.014) analyses supported significant differences in gut microbial community structure between the two groups. Discriminating taxa associated with sarcopenia in UC were identified by LEfSe at the genus level. *Bacteroides*, *Podoviridae*, and *Porphyromonas* were significantly depleted in the gut microbiota of the sarcopenia group, whereas *Streptococcus*, *Catenibacterium, Methanosphaera,* and *Lactococcus* were significantly enriched (Figure 4B).

The gut microbial community composition of either sarcopenic UC patients or non-sarcopenic UC patients differed significantly from that of HCs, as evidenced by results of ANOSIM (Supplementary Figure 1) and MRPP (Supplementary Table 3). LEfSe analysis between UC patients with sarcopenia and HCs also showed that *Bacteroides* was significantly decreased, while *Streptococcus* was significantly increased, in the sarcopenia group. However, *Bacteroides* and *Streptococcus* were not among the distinguishing genera between UC patients without sarcopenia and HCs. The LEfSe results of gut microbiota between UC patients with or without sarcopenia and HCs are exhibited in Supplementary Figures 2, 3, respectively.

### 3.7 Associations between sarcopenia and serum metabolites or gut microbiota in UC patients

Whether the altered serum metabolites and gut bacterial taxa in sarcopenic UC patients were significantly related to sarcopenia, independent of disease severity and systemic inflammation, was assessed. After adjusting for disease activity and serum hsCRP, GCDCA-3S, GCA, primary BAs, conjugated BAs, conjugated primary BAs, and *Streptococcus* remained positively associated with sarcopenia (all *p* < 0.05), whereas valerylcarnitine and *Bacteroides* remained negatively associated with sarcopenia (both *p* < 0.05). A borderline significant negative association was observed between 2-methylbutyrylcarnitine and sarcopenia (*p* = 0.049). However, TCA and hippuric acid lost their significant associations with sarcopenia in UC (Table 4).

**Table 4:** Adjusted associations between sarcopenia and serum metabolites or gut microbiota in UC patients.

Variables	OR	95% CrI	*p* value
GCDCA-3S	8.18	2.91–33.90	< 0.001
GCA	2.35	1.15–6.64	0.017
TCA	1.25	0.71–2.38	0.433
Primary BAs	2.02	1.10–4.44	0.023
Conjugated BAs	2.58	1.31–6.07	0.005
Conjugated primary BAs	2.47	1.25–5.97	0.008
Hippuric acid	0.60	0.23–1.18	0.165
Valerylcarnitine	0.27	0.10–0.61	0.003
2-Methylbutyrylcarnitine	0.54	0.26–0.99	0.049
*Bacteroides*	0.37	0.14–0.76	0.004
*Streptococcus*	8.60	2.08–71.59	0.002

Models were adjusted for disease activity and serum hsCRP. OR, odds ratio; CrI, credible interval; GCDCA-3S, glycochenodeoxycholic acid-3-sulfate; GCA, glycocholic acid; TCA, taurocholic acid; BAs, bile acids.

### 3.8 KEGG functional analysis of the gut microbiota

**Figure 5:**
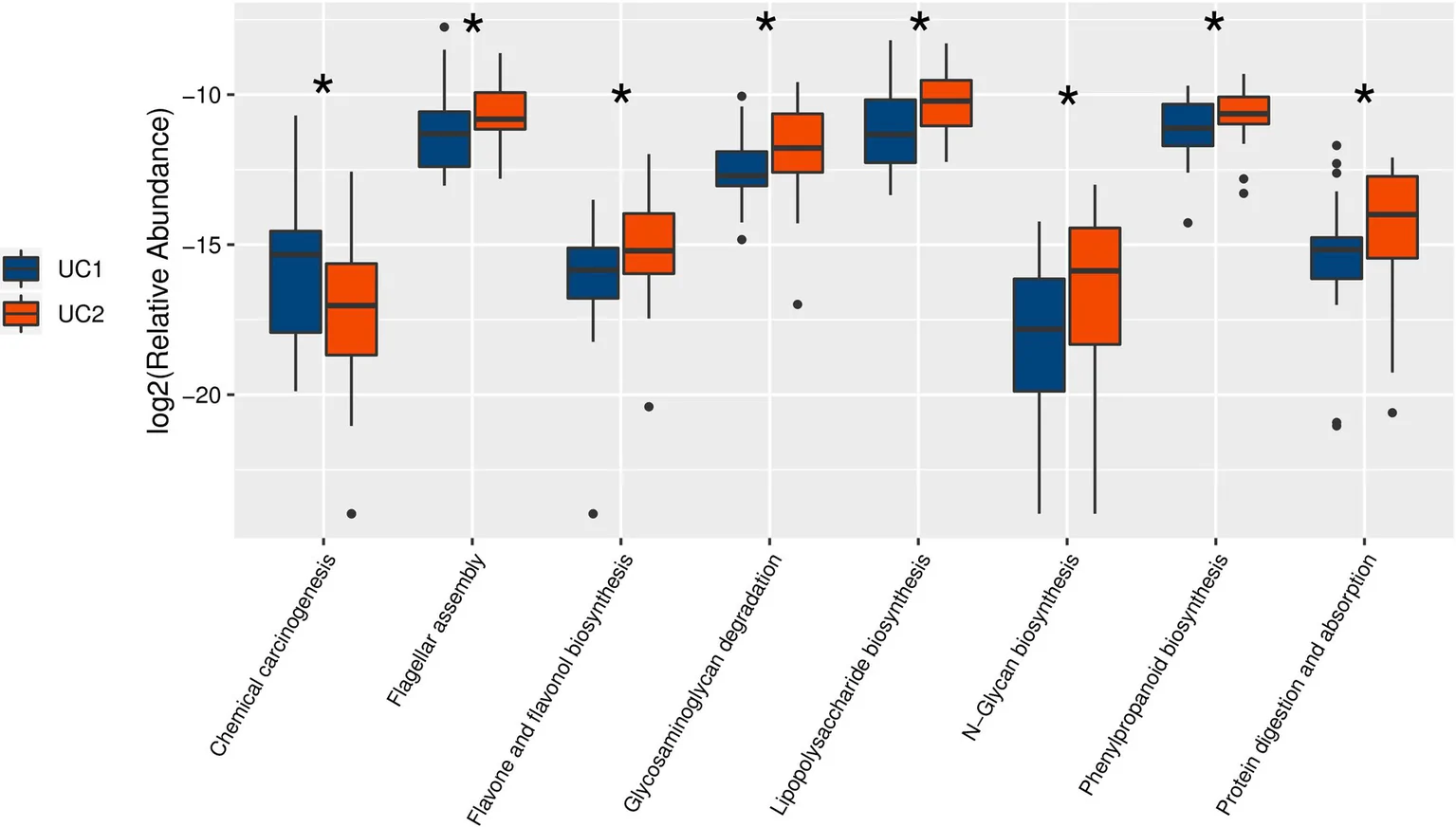
Differential microbial-affiliated metabolic pathways in sarcopenic and non-sarcopenic UC patients. *p < 0.05.

**Figure 6:**
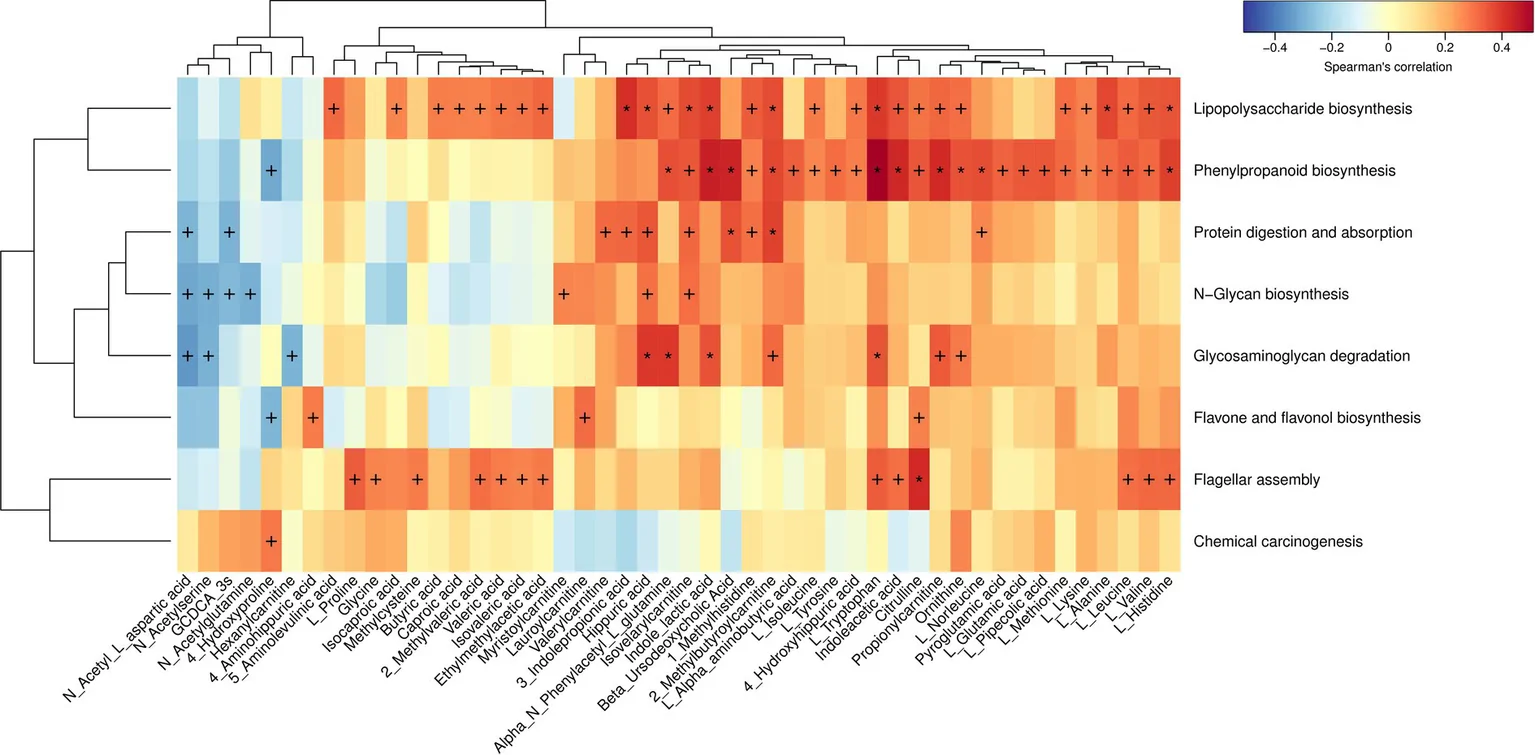
Correlations between the relative abundance of microbial-affiliated metabolic pathways and the serum concentration of metabolites in UC patients. ^+^*p* < 0.05; *p < 0.01.

KEGG functional analysis revealed that the relative abundance of several pathways was significantly lower in UC patients with sarcopenia compared with those without sarcopenia (Figure 5). Notably, the protein digestion and absorption pathway was significantly less abundant in sarcopenic UC patients (*p* = 0.030). Furthermore, the pathway related to secondary bile acid biosynthesis tended to be less abundant in UC patients with sarcopenia, although statistical significance was not reached (*p* = 0.096). Correlations between the relative abundance of gut microbial functional pathways and serum metabolite concentrations across the entire UC cohort are depicted in Figure 6. The protein digestion and absorption pathway exhibited significant positive correlations with serum valerylcarnitine (*r* = 0.313, *p* = 0.020), hippuric acid (*r* = 0.344, *p* = 0.010), isovalerylcarnitine (*r* = 0.315, *p* = 0.019), and 2-methylbutyrylcarnitine (*r* = 0.377, *p* = 0.005), and a significant negative correlation with GCDCA-3S (*r* = −0.292, *p* = 0.031).

## 4 Discussion

In this study, we demonstrated a high prevalence of sarcopenia in hospitalized patients with active UC according to the updated AWGS 2025 criteria. Sarcopenia was associated with systemic inflammation, alterations in circulating bile acids and acylcarnitines, gut dysbiosis, and predicted functional changes in microbial metabolic pathways.

Given the high cost of magnetic resonance imaging (MRI), the radiation exposure associated with whole-body CT, and the limited accessibility of dual-energy X-ray absorptiometry (DXA), the most widely employed measurement methods in both clinical practice and research are BIA and CT of the thoracic or lumbar muscle region. The present study demonstrated that L3-SMI measured by CT correlated well with ASMI and TSMI measured by BIA, and L3-SMI was also significantly positively correlated with handgrip strength and PhA. With respect to adiposity assessment, CT-derived L3-SFA correlated well with BIA-measured FM and FM%, and VFA values derived from the two methods were also significantly positively correlated. These findings indicate that, in settings where BIA equipment is unavailable, indices of muscle and subcutaneous fat at the L3 cross-section measured by CT can serve as reliable surrogates for muscle and fat mass in UC patients. However, owing to the absence of a universally accepted reference for CT-derived measurements, the identification of low muscle mass using CT remains heterogeneous across studies (25).

Although the AWGS and EWGSOP consensus statements mainly focus on age-related sarcopenia, the diagnostic cut-off values for BIA-measured muscle parameters provide an approach for assessing muscle status in chronic disease-related sarcopenia. To the best of our knowledge, this is the first study to report the prevalence of sarcopenia in UC patients according to the AWGS 2025 criteria. In our study, the prevalence of sarcopenia was 40.4% by AWGS 2025 versus only 17.5% by AWGS 2019, the latter of which is consistent with a previous report of 13.1% in hospitalized UC patients based on AWGS 2019 (26). We found that an additional 11 male patients were identified as having sarcopenia by the AWGS 2025 criteria compared with AWGS 2019, and the prevalence of sarcopenia and low muscle strength in patients aged 18–49 years was significantly higher under the AWGS 2025 criteria than the AWGS 2019 criteria. These results indicate that the less stringent AWGS 2025 thresholds for the middle-aged population may facilitate the identification of sarcopenia in UC patients (particularly in males), given that UC predominantly affects young and middle-aged individuals. However, it should also be noted that the AWGS 2025 criteria may result in overdiagnosis of sarcopenia in the UC population. In addition, we did not find a higher prevalence of sarcopenia in older UC patients than in younger UC patients under either criteria, which further suggested a risk of overestimating sarcopenia prevalence in younger UC patients when these criteria are applied. The prevalence of sarcopenia based on AWGS 2025 cut-off values should be verified in studies with larger sample sizes, and further longitudinal research is warranted to examine the association between sarcopenia diagnosed according to AWGS 2025 and clinical outcomes in UC, thereby clarifying the clinical relevance and applicability of these criteria in the UC population.

In the present study, female patients exhibited significant reductions in both TSMI and ASMI, whereas male patients exhibited a significant reduction only in TSMI. Considering that no significant differences in disease duration, disease activity, or serum inflammatory markers were found between male and female patients, the distinct patterns of muscle loss in male and female patients cannot be simply attributed to differences in disease-related factors. Our previous retrospective study of 457 patients with active UC identified a higher rate of myopenia in males (52.0%) than in females (46.8%) when determined by CT-derived L3-SMI, which reflects truncal muscle mass (21); in contrast, the present study found a lower rate of myopenia in male patients (43.8%) than in female patients (60.0%) when assessed by ASMI, indicating that TSMI and ASMI are not fully interchangeable measures of muscle mass. Assessment of both truncal and appendicular muscle mass may provide a more comprehensive evaluation of muscle mass in IBD patients.

Diamine oxidase (DAO), a highly active intracellular enzyme synthesized in intestinal mucosal villi, is released into the systemic circulation upon intestinal mucosal injury and disruption of epithelial barrier integrity. Because serum DAO activity remains relatively stable, it has been used as a sensitive and reliable biomarker for evaluating intestinal barrier damage and restoration in IBD (27, 28). In the present study, no significant difference was observed in serum DAO between UC patients with and without sarcopenia, though UC patients with myopenia or low muscle strength tended to have higher serum DAO concentration. Theoretically, impaired intestinal barrier function can perpetuate insulin resistance and adversely affect mitochondrial function in skeletal muscle, thereby precluding muscle cells from effective utilization of glucose and promoting muscle protein breakdown (29, 30). However, our findings indicate that a confirmed association between intestinal barrier function and sarcopenia in UC still requires more evidence.

The present study demonstrated that serum conjugated BAs, particularly conjugated primary BAs, were significantly elevated in sarcopenic UC patients compared with non-sarcopenic UC patients, whereas the differences in these BAs between non-sarcopenic UC patients and HCs were not significant, suggesting a close relationship between BAs and sarcopenia in UC. Although data on the role of BAs in muscle health in IBD are sparse, evidence from chronic liver disease (CLD) supports a deleterious effect of conjugated BAs on skeletal muscle. A recent study reported that serum tauro-conjugated BA profiles were inversely correlated with muscle cross-sectional area in patients with CLD, and GCDCA exhibited a negative correlation with the fast-twitch marker MYH4 in C2C12 myotubes, promoting a shift from fast to slow muscle fiber types and contributing to muscle loss (31). Moreover, preclinical research has shown increased conjugated BAs in the liver and serum of cancer cachexia mouse models, likely attributable to enhanced hepatic conjugation of primary and secondary BAs to taurine and glycine, coupled with reduced intestinal BA deconjugation (32).

*Bacteroides* and *Streptococcus*, as the notably changed genera in sarcopenic UC patients in this study, have received attention in prior sarcopenia research. Studies from Japan demonstrated that the relative abundance of *Streptococcus* was significantly negatively correlated with grip strength, whereas *Bacteroides* was significantly positively correlated with leg extension power (33, 34). Interestingly, *Bacteroides* and *Streptococcus* exhibit divergent metabolic capacities toward BAs. The deconjugation of tauro- and glyco-conjugated BAs depends on bile salt hydrolase (BSH) enzymes produced by specific gut microbes. *Bacteroides* species are among the bacteria with high BSH activity (35, 36), whereas *Streptococcus* species are predominantly adapted to dairy and plant environments rather than the bile-rich intestinal habitat and generally lack BSH activity (37). In parallel, the conversion of primary BAs to secondary BAs is also microbiota-dependent. The significantly elevated serum primary BAs in UC patients with sarcopenia might result from compromised secondary BA biosynthesis by the gut microbiota, which was supported by the KEGG functional analysis in this study, leaving more primary BAs in the colon and then absorbed into the blood. Collectively, the findings in the present study suggest that sarcopenia in UC might be linked to dysregulated BA metabolism and gut dysbiosis.

Although we found a significantly lower level of serum hippuric acid in sarcopenic UC patients, the associations between sarcopenia and serum hippuric acid lost significance after adjusting for disease activity and hsCRP levels. The decreased level of urinary hippuric acid in IBD patients has been reported in a systematic review of metabolomic analysis in IBD (38), and accordingly, the decreased concentration of circulating hippuric acid has also been observed in IBD patients (39). Our findings suggested that the alteration in hippuric acid metabolism may be associated with UC rather than UC-related sarcopenia.

With respect to acylcarnitines, we observed that the serum concentrations of valerylcarnitine and 2-methylbutyrylcarnitine showed a stepwise increase among sarcopenic UC patients, non-sarcopenic UC patients, and HCs, and their associations with sarcopenia remained significant after adjusting for disease activity and hsCRP. Data on circulating acylcarnitine levels in UC patients are limited. A previous study demonstrated lower plasma isovalerylcarnitine in UC patients than in healthy controls (40), while decreased valerylcarnitine or 2-methylbutyrylcarnitine has not been reported in these patients. Acylcarnitines can modulate muscle energy metabolism and the whole-body energy balance (41). Valerylcarnitine belongs to the short-chain acylcarnitines (C2–C5), primarily derived from fatty acid β-oxidation, whereas isovalerylcarnitine and 2-methylbutyrylcarnitine are branched-chain acylcarnitines derived from the catabolism of the BCAAs leucine and isoleucine, respectively (42). Research in older females has shown that low intramuscular short-chain acylcarnitine levels correlate with reduced physical performance and decreased expression of genes involved in mitochondrial energy production and functionality (43). The findings of the present study suggest that the roles of valerylcarnitine and 2-methylbutyrylcarnitine in the muscle health of UC patients deserve further attention in future research.

KEGG functional analysis in this study showed decreased protein digestion and absorption pathways in the gut microbial community of sarcopenic UC patients. Certain bacterial species are capable of *de novo* biosynthesis of essential amino acids and participate in regulating the host amino acid pool (44, 45). Moreover, the gut microbiota can modulate amino acid bioavailability by utilizing various amino acids derived from dietary and endogenous proteins, thereby influencing protein synthesis and degradation in the host’s skeletal muscle (46, 47). Interestingly, significant positive correlations were found between the level of protein digestion and absorption pathways and serum valerylcarnitine, 2-methylbutyrylcarnitine, and isovalerylcarnitine. The intersection of acylcarnitines and protein metabolism may involve BCAAs (48). These findings suggest that compromised participation of the gut microbiota in protein digestion and absorption might be involved in sarcopenia in UC patients, which requires further exploration in pre-clinical research.

The present study has several limitations. First, owing to the observational, cross-sectional design, causal inferences cannot be drawn from our results. A longitudinal study design would provide the opportunity to establish causality between dysbiosis, metabolic alterations, and sarcopenia in UC, and future animal experiments involving fecal microbiota transplantation or genus-specific interventions are necessary to explore the underlying mechanisms. Second, the data from a single-center cohort with a relatively small sample size are not sufficiently representative of the UC population, limiting the generalizability of the findings, especially to UC patients in the outpatient clinic. Additionally, the high proportion of patients with severe disease activity in our hospitalized cohort may have led to a relatively high estimated prevalence of sarcopenia. Therefore, multicenter studies with larger sample sizes are required to verify the prevalence of sarcopenia in UC patients. Third, no dietary or pharmacological control was implemented in this study, although both factors are important potential confounders that may influence body composition, gut microbiota, and circulating metabolites. Detailed dietary data should be collected in future studies, and data analysis should be optimized by stratifying according to therapeutic drug categories. Finally, because the present study is exploratory and hypothesis-generating rather than confirmatory, we did not apply a formal correction for multiple comparisons (49). Nevertheless, the risk of false-positive findings may be increased, and our results warrant further validation in the future.

## 5 Conclusion

The prevalence of sarcopenia was 40.4% in this UC cohort. Sarcopenia in younger UC patients can be identified more sensitively using the AWGS 2025 criteria compared with AWGS 2019. UC-related sarcopenia may be associated with systemic inflammation, gut dysbiosis, especially decreased *Bacteroides* and increased *Streptococcus*, and altered circulating metabolites, including BAs and acylcarnitines.

## Data Availability

The original contributions presented in the study are publicly available. This data can be found in the NCBI repository: https://www.ncbi.nlm.nih.gov/bioproject/PRJNA1499619.

## Supplementary material

SUPPLEMENTARY FIGURE 1 Analysis of Similarities (ANOSIM) showed that the gut microbial community composition of either sarcopenic UC patients or non-sarcopenic UC patients differed significantly from that of healthy controls. **(A)** The results for sarcopenic UC patients and healthy controls. **(B)** The results for non-sarcopenic UC patients and healthy controls. UC1, UC patients with sarcopenia; UC2, UC patients without sarcopenia; HC, healthy controls.

SUPPLEMENTARY FIGURE 2 The distinguishing taxa at the genus level between sarcopenic UC patients and healthy controls. UC1, UC patients with sarcopenia; HC, healthy controls.

SUPPLEMENTARY FIGURE 3 The distinguishing taxa at the genus level between non-sarcopenic UC patients and healthy controls. UC2, UC patients without sarcopenia; HC, healthy controls.

## References

[1] Ma T Wan M Liu G Zuo X Yang X Yang X. Temporal trends of inflammatory bowel disease burden in China from 1990 to 2030 with comparisons to Japan, South Korea, the European Union, the United States of America, and the world. Clin Epidemiol. (2023) 15:583–99. doi: 10.2147/CLEP.S402718, 37187768 PMC10178411

[2] Yang H Qian J. Epidemiological research, burden, and clinical advances of inflammatory bowel disease in China. Chin Med J. (2024) 137:1009–11. doi: 10.1097/cm9.0000000000003064, 38704618 PMC11062669

[3] Le Berre C Honap S Peyrin-Biroulet L. Ulcerative colitis. Lancet. (2023) 402:571–84. doi: 10.1016/S0140-6736(23)00966-2, 37573077

[4] Glassner KL Abraham BP Quigley EMM. The microbiome and inflammatory bowel disease. J Allergy Clin Immunol. (2020) 145:16–27. doi: 10.1016/j.jaci.2019.11.003, 31910984

[5] Zhang YZ Li YY. Inflammatory bowel disease: pathogenesis. World J Gastroenterol. (2014) 20:91–9. doi: 10.3748/wjg.v20.i1.91, 24415861 PMC3886036

[6] Calvez V Becherucci G Covello C Piccirilli G Mignini I Esposto G . Navigating the intersection: sarcopenia and Sarcopenic obesity in inflammatory bowel disease. Biomedicine. (2024) 12:1218. doi: 10.3390/biomedicines12061218, 38927425 PMC11200968

[7] Nakase H Sato N Mizuno N Ikawa Y. The influence of cytokines on the complex pathology of ulcerative colitis. Autoimmun Rev. (2022) 21:103017. doi: 10.1016/j.autrev.2021.103017, 34902606

[8] Troisi S Sicilia G Petito V Masi L Deleu S Migliore G . The molecular basis of sarcopenia in inflammatory bowel disease: from gut-muscle axis to therapeutic opportunities. Minerva Gastroenterol. (2025) 71:351–69. doi: 10.23736/S2724-5985.25.03967-1, 41263530

[9] Fatani H Olaru A Stevenson R Alharazi W Jafer A Atherton P . Systematic review of sarcopenia in inflammatory bowel disease. Clin Nutr. (2023) 42:1276–91. doi: 10.1016/j.clnu.2023.05.002, 37352818

[10] Neelam PB Pal R Gupta P Singh AK Shah J Mandavdhare HS . Sarcopenia is common in ulcerative colitis and correlates with disease activity. Intest Res. (2024) 22:162–71. doi: 10.5217/ir.2023.00090, 38247117 PMC11079510

[11] Feng Y Feng W Xu M Wu C Yang H Wang Y . Sarcopenia and treatment failure in inflammatory bowel disease: a systematic review and meta-analysis. Rev Esp Enferm Dig. (2024) 116:68–76. doi: 10.17235/reed.2023.9808/2023, 37706492

[12] Ge X Jiang L Yu W Wu Y Liu W Qi W . The importance of sarcopenia as a prognostic predictor of the clinical course in acute severe ulcerative colitis patients. Dig Liver Dis. (2021) 53:965–71. doi: 10.1016/j.dld.2021.03.031, 33934998

[13] Ticinesi A Lauretani F Milani C Nouvenne A Tana C Del Rio D . Aging gut microbiota at the cross-road between nutrition, physical frailty, and sarcopenia: is there a gut-muscle axis? Nutrients. (2017) 9:1303. doi: 10.3390/nu9121303, 29189738 PMC5748753

[14] Liang Y Lu C Ma D He X. Gut microbiome mediates the effect of inflammatory bowel disease on sarcopenia: a bidirectional Mendelian randomization study. Digestion. (2025) Epub ahead of print. 1–11. doi: 10.1159/000549749, 41317335

[15] Deng B Liu Y He P Wang N Xu L Ma J. Shared molecular features and potential diagnostic biomarkers between ulcerative colitis and sarcopenia: an integrative bioinformatics analysis with preliminary experimental validation. Front Immunol. (2026) 17:1801914. doi: 10.3389/fimmu.2026.1801914, 42064059 PMC13124693

[16] Picca A Ponziani FR Calvani R Marini F Biancolillo A Coelho-Junior HJ . Gut microbial, inflammatory and metabolic signatures in older people with physical frailty and sarcopenia: results from the BIOSPHERE study. Nutrients. (2020) 12:65. doi: 10.3390/nu12010065, 31887978 PMC7019826

[17] Jager R Zaragoza J Purpura M Iametti S Marengo M Tinsley GM . Probiotic administration increases amino acid absorption from plant protein: a placebo-controlled, randomized, double-blind, multicenter, crossover study. Probiotics Antimicrob Proteins. (2020) 12:1330–9. doi: 10.1007/s12602-020-09656-5, 32358640 PMC7641926

[18] Wang Z Shi Z Song Q Lu W Sun Z Zang J . Altered serum short-chain fatty acids in sarcopenia among Chinese elderly women: a case-control study. BMC Geriatr. (2025) 25:1018. doi: 10.1186/s12877-025-06564-7, 41398907 PMC12706993

[19] Magro F Gionchetti P Eliakim R Ardizzone S Armuzzi A Barreiro-de Acosta M . Third European evidence-based consensus on diagnosis and management of ulcerative colitis. Part 1: definitions, diagnosis, extra-intestinal manifestations, pregnancy, cancer surveillance, surgery, and ileo-anal pouch disorders. J Crohns Colitis. (2017) 11:649–70. doi: 10.1093/ecco-jcc/jjx008, 28158501

[20] Chen L-K Hsiao F-Y Akishita M Assantachai P Lee W-J Lim WS . A focus shift from sarcopenia to muscle health in the Asian working Group for Sarcopenia 2025 consensus update. Nat Aging. (2025) 5:2164–75. doi: 10.1038/s43587-025-01004-y, 41188603

[21] Wei W Yan P Zhang Y Wang Q Kang J Liu P . Myopenia and body fat distribution in hospitalized ulcerative colitis patients: correlations with clinical characteristics and response to vedolizumab. Front Nutr. (2024) 11:1411695. doi: 10.3389/fnut.2024.1411695, 39758314 PMC11695233

[22] Chen L-K Woo J Assantachai P Auyeung T-W Chou M-Y Iijima K . Asian working Group for Sarcopenia: 2019 consensus update on sarcopenia diagnosis and treatment. J Am Med Dir Assoc. (2020) 21:300–307.e2. doi: 10.1016/j.jamda.2019.12.012, 32033882

[23] Gelman A Jakulin A Pittau MG Su Y-S. A weakly informative default prior distribution for logistic and other regression models. Ann Appl Stat. (2008) 2:1360–83. doi: 10.1214/08-aoas191

[24] Greenland S Mansournia MA Altman DG. Sparse data bias: a problem hiding in plain sight. BMJ. (2016) 352:i1981. doi: 10.1136/bmj.i1981, 27121591

[25] Su H Ruan J Chen T Lin E Shi L. CT-assessed sarcopenia is a predictive factor for both long-term and short-term outcomes in gastrointestinal oncology patients: a systematic review and meta-analysis. Cancer Imaging. (2019) 19:82. doi: 10.1186/s40644-019-0270-0, 31796090 PMC6892174

[26] Zhang Y Zhang L Gao X Dai C Huang Y Wu Y . Impact of malnutrition and sarcopenia on quality of life in patients with inflammatory bowel disease: a multicentre study. J Cachexia Sarcopenia Muscle. (2023) 14:2663–75. doi: 10.1002/jcsm.13341, 37779327 PMC10751433

[27] Song W-B Lv Y-H Zhang Z-S Li Y-N Xiao L-P Yu X-P . Soluble intercellular adhesion molecule-1, D-lactate and diamine oxidase in patients with inflammatory bowel disease. World J Gastroenterol. (2009) 15:3916–9. doi: 10.3748/wjg.15.3916, 19701972 PMC2731254

[28] Cai J Chen H Weng M Jiang S Gao J. Diagnostic and clinical significance of serum levels of D-lactate and diamine oxidase in patients with Crohn’s disease. Gastroenterol Res Pract. (2019) 2019:1–7. doi: 10.1155/2019/8536952, 31531016 PMC6721431

[29] Grosicki GJ Fielding RA Lustgarten MS. Gut microbiota contribute to age-related changes in skeletal muscle size, composition, and function: biological basis for a gut-muscle axis. Calcif Tissue Int. (2018) 102:433–42. doi: 10.1007/s00223-017-0345-5, 29058056 PMC5858871

[30] Zhao J Huang Y Yu X. A narrative review of gut-muscle Axis and sarcopenia: the potential role of gut microbiota. Int J Gen Med. (2021) 14:1263–73. doi: 10.2147/ijgm.S301141, 33880058 PMC8053521

[31] Iwasa M Eguchi A Kohjima M Miyazaki T Kitamura H Takami Y . The conjugated bile acids profile suggests a novel liver-muscle axis associated with sarcopenia in chronic liver disease. Liver Int. (2026) 46:e70612. doi: 10.1111/liv.70612, 41902587 PMC13032176

[32] Feng L Zhang W Shen Q Miao C Chen L Li Y . Bile acid metabolism dysregulation associates with cancer cachexia: roles of liver and gut microbiome. J Cachexia Sarcopenia Muscle. (2021) 12:1553–69. doi: 10.1002/jcsm.12798, 34585527 PMC8718071

[33] Yasuda T Takagi T Naito Y Inoue R Mizushima K Asaeda K . Sarcopenia-related gut microbiota in the elderly: insights from the longevity region of Kyotango and its nutritional associations. Gut Microbes Rep. (2025) 2:2591561. doi: 10.1080/29933935.2025.2591561, 41909896 PMC12940105

[34] Iwasaka C Nanri H Nakagata T Ohno H Tanisawa K Konishi K . Association of skeletal muscle function, quantity, and quality with gut microbiota in Japanese adults: a cross-sectional study. Geriatr Gerontol Int. (2024) 24:53–60. doi: 10.1111/ggi.14751, 38098315

[35] Guimarães Silva CM Ferreira RBR Lobo LA. Microbial adaptation and host signaling: Bacteroides-bile acid interactions in gut health. Anaerobe. (2026) 98:103041. doi: 10.1016/j.anaerobe.2026.103041, 41966474

[36] Li W Chen H Tang J. Interplay between bile acids and intestinal microbiota: regulatory mechanisms and therapeutic potential for infections. Pathogens. (2024) 13:702. doi: 10.3390/pathogens13080702, 39204302 PMC11356816

[37] Tanaka H Doesburg K Iwasaki T Mierau I. Screening of lactic acid bacteria for bile salt hydrolase activity. J Dairy Sci. (1999) 82:2530–5. doi: 10.3168/jds.S0022-0302(99)75506-2 10629797

[38] Gallagher K Catesson A Griffin JL Holmes E Williams HRT. Metabolomic analysis in inflammatory bowel disease: a systematic review. J Crohns Colitis. (2021) 15:813–26. doi: 10.1093/ecco-jcc/jjaa227, 33175138

[39] Wu X Liu K Wu Q Wang M Chen X Li Y . Biomarkers of metabolomics in inflammatory bowel disease and damp-heat syndrome: a preliminary study. Evid Based Complement Alternat Med. (2022) 2022:3319646. doi: 10.1155/2022/3319646, 35815273 PMC9270137

[40] Bene J Komlósi K Havasi V Talián G Gasztonyi B Horváth K . Changes of plasma fasting carnitine ester profile in patients with ulcerative colitis. World J Gastroenterol. (2006) 12:110–3. doi: 10.3748/wjg.v12.i1.110, 16440427 PMC4077497

[41] Xiang F Zhang Z Xie J Xiong S Yang C Liao D . Comprehensive review of the expanding roles of the carnitine pool in metabolic physiology: beyond fatty acid oxidation. J Transl Med. (2025) 23:324. doi: 10.1186/s12967-025-06341-5, 40087749 PMC11907856

[42] Dambrova M Makrecka-Kuka M Kuka J Vilskersts R Nordberg D Attwood MM . Acylcarnitines: nomenclature, biomarkers, therapeutic potential, drug targets, and clinical trials. Pharmacol Rev. (2022) 74:506–51. doi: 10.1124/pharmrev.121.000408, 35710135

[43] van der Hoek MD Nieuwenhuizen AG Kuda O Bos P Paluchova V Verschuren L . Intramuscular short-chain acylcarnitines in elderly people are decreased in (pre-)frail females, but not in males. FASEB J. (2020) 34:11658–71. doi: 10.1096/fj.202000493R, 32672378

[44] Dai ZL Wu G Zhu WY. Amino acid metabolism in intestinal bacteria: links between gut ecology and host health. Front Biosci. (2011) 16:1768–86. doi: 10.2741/3820, 21196263

[45] Lin R Liu W Piao M Zhu H. A review of the relationship between the gut microbiota and amino acid metabolism. Amino Acids. (2017) 49:2083–90. doi: 10.1007/s00726-017-2493-3, 28932911

[46] Neis EP Dejong CH Rensen SS. The role of microbial amino acid metabolism in host metabolism. Nutrients. (2015) 7:2930–46. doi: 10.3390/nu7042930, 25894657 PMC4425181

[47] Karlund A Gomez-Gallego C Turpeinen AM Palo-Oja OM El-Nezami H Kolehmainen M. Protein supplements and their relation with nutrition, microbiota composition and health: is more protein always better for sportspeople? Nutrients. (2019) 11:829. doi: 10.3390/nu11040829, 31013719 PMC6521232

[48] McCann MR George De la Rosa MV Rosania GR Stringer KA. L-carnitine and acylcarnitines: mitochondrial biomarkers for precision medicine. Metabolites. (2021) 11:51. doi: 10.3390/metabo11010051, 33466750 PMC7829830

[49] Bender R Lange S. Adjusting for multiple testing--when and how? J Clin Epidemiol. (2001) 54:343–9. doi: 10.1016/s0895-4356(00)00314-0 11297884

