# Supplementary material for: Sarcopenia in patients with active ulcerative colitis: associations with serum metabolites and gut microbiota
Source: Front Nutr. 2026 Sep 3;13:1942104. doi: 10.3389/fnut.2026.1942104 (PMC13581694; doi:10.3389/fnut.2026.1942104)
Supplement: Supplementary file 1 [file Table_1.docx]

**Table S1 Serum metabolites measured in this study**

| Amino Acids | L-Lysine | Bile Acids | Cholic acid (CA) |
| --- | --- | --- | --- |
|  | L-Histidine |  | Chenodeoxycholic acid (CDCA) |
|  | L-Arginine |  | Deoxycholic acid (DCA) |
|  | Ornithine |  | Lithocholic acid (LCA) |
|  | L-Glutamine |  | isolithocholic acid (isoLCA) |
|  | L-Glutamic acid |  | Beta-Ursodeoxycholic Acid (β-UDCA） |
|  | L-Alanine |  | Glycocholic acid (GCA) |
|  | Dimethylglycine |  | Glycochenodeoxycholic acid (GCDCA) |
|  | L-Serine |  | Glycodeoxycholic acid (GDCA) |
|  | L-threonine |  | Glycolithocholic Acid-3-Sulfate (GLCA-3S) |
|  | Creatine |  | GCDCA-3S |
|  | Methylcysteine |  | GDCA-3S |
|  | L-Tyrosine |  | Glycoursodeoxycholic acid (GUDCA) |
|  | L-Asparagine |  | Taurocholic acid (TCA) |
|  | L-Phenylalanine |  | Taurochenodeoxycholic acid (TCDCA) |
|  | L-Kynurenine |  | Taurodeoxycholic acid (TDCA) |
|  | L-Aspartic acid |  | Taurolithocholic acid (TLCA) |
|  | N-Acetyl-L-aspartic acid |  | Tauroursodeoxycholic acid (TUDCA) |
|  | Glycine | Carnitines | Carnitine |
|  | Citrulline |  | L_Acetylcarnitine |
|  | L-Alpha-aminobutyric acid |  | Propionylcarnitine |
|  | L-Proline |  | Butyrylcarnitine |
|  | Acetylglycine |  | 2_Methylbutyroylcarnitine |
|  | L-Pipecolic acid |  | Valerylcarnitine |
|  | N-Acetylserine |  | Isovelarylcarnitine |
|  | N-Acetylglutamine |  | 3_Hydroxylisovalerylcarnitine |
|  | L-Valine |  | Hexanylcarnitine |
|  | Pyroglutamic acid |  | Octanoylcarnitine |
|  | 5-Aminolevulinic acid |  | Decanoylcarnitine C10 |
|  | L-Methionine |  | Lauroylcarnitine |
|  | L-Isoleucine |  | Myristoylcarnitine |
|  | L-Leucine |  | Palmitoylcarnitine |
|  | L-Norleucine |  | Oleylcarnitine C18 1 |
|  | L-Tryptophan |  | Linoleylcarnitine |
|  | Alpha-N-Phenylacetyl-L-glutamine |  | Stearylcarnitine |
|  | 1-Methylhistidine | SCFAs | Acetic acid |
|  | 4-Hydroxyproline |  | 2-Methylacetic acid |
| Benzoic Acids | 4-Hydroxyhippuric acid |  | Propanoic acid |
|  | 4-Hydroxybenzoic acid |  | Butyric acid |
|  | Hippuric acid |  | Isobutyric acid |
|  | 4-Aminohippuric acid |  | Isovaleric acid |
|  | Phthalic acid |  | Valeric acid |
| Indoles | Indoleacetic acid |  | 2-Methylvaleric acid |
|  | 3-Indolepropionic acid |  | Isocaproic acid |
|  | Indole-lactic acid |  | Caproic acid |

**Table S2 UPLC-MS/MS instrument settings**

| UPLC | |
| --- | --- |
| Column | ACQUITY UPLC BEH C18 1.7 µM VanGuard pre-column(2.1×5 mm) and ACQUITY UPLC BEH C18 1.7 µM analytical column (2.1 × 100 mm) |
| Column Temp. (°C) | 40 |
| Sample Manager Temp.(°C) | 10 |
| Mobile Phases | A=water with 0.1% formic acid; and B=acetonitrile / IPA(70:30) |
| Gradient Conditions | 0-1 min (5% B), 1-11min (5-78% B), 11-13.5 min (78-95%B), 13.5-14 min (95-100% B), 14-16 min (100% B)，16-16.1min (100-5% B)，16.1-18 min (5% B). |
| Flow Rate (mL/min) | 0.40 |
| Injection Vol. (µl) | 5.0 |
| Mass Spectrometer | |
| Capillary (Kv) | 1.5 (ESI+), 2.0 (ESI-) |
| Source Temp (°C) | 150 |
| Desolvation Temp (°C) | 550 |
| Desolvation Gas Flow (L/Hr) | 1000 |

**Table S3 Multi-Response Permutation Procedure analysis of gut microbiota between ulcerative colitis patients and healthy controls**

|  | A value | *p* value |
| --- | --- | --- |
| HC-UC1 | 0.081 | 0.001 |
| HC-UC2 | 0.090 | 0.001 |

UC1: UC patients with sarcopenia; UC2: UC patients without sarcopenia; HC: healthy controls.
